# Impact of diabetes mellitus on ischemic cardiomyopathy. Five-year follow-up. REVISION-DM trial

**DOI:** 10.1186/s13098-018-0320-y

**Published:** 2018-03-15

**Authors:** Thiago Hueb, Mauricio S. Rocha, Sergio F. Siqueira, Silvana Angelina D´Orio Nishioka, Giselle L. Peixoto, Marcos M. Saccab, Eduardo Gomes Lima, Rosa Maria Rahmi Garcia, José Antonio F. Ramires, Roberto Kalil Filho, Martino Martinelli Filho

**Affiliations:** Clinical Division, Heart Institute (InCor) of the University of São Paulo, Av. Dr. Eneas de Carvalho Aguiar 44, AB Cerqueira César, São Paulo, SP 05403–000 Brazil

**Keywords:** Ischemic cardiomyopathy, Ventricular dysfunction, Diabetes mellitus, Coronary artery disease

## Abstract

**Background:**

Patients with ischemic cardiomyopathy and severe left ventricular dysfunction have a worse survival prognosis than patients with preserved ventricular function. The role of diabetes in the long-term prognosis of this patient group is unknown. This study investigated whether the presence of diabetes has a long-term impact on left ventricular function.

**Methods:**

Patients with coronary artery disease who underwent coronary artery bypass graft surgery, percutaneous coronary intervention, or medical therapy alone were included. All patients had multivessel disease and left ventricular ejection fraction measurements. Overall mortality, nonfatal myocardial infarction, stroke, and additional interventions were investigated.

**Results:**

From January 2009 to January 2010, 918 consecutive patients were selected and followed until May 2015. They were separated into 4 groups: G1, 266 patients with diabetes and ventricular dysfunction; G2, 213 patients with diabetes without ventricular dysfunction; G3, 213 patients without diabetes and ventricular dysfunction; and G4, 226 patients without diabetes but with ventricular dysfunction. Groups 1, 2, 3, and 4, respectively, had a mortality rate of 21.6, 6.1, 4.2, and 10.6% (*P* < .001); nonfatal myocardial infarction of 5.3, .5, 7.0, and 2.6% (*P* < .001); stroke of .40, .45, .90, and .90% (*P* = NS); and additional intervention of 3.8, 11.7, 10.3, and 2.6% (*P* < .001).

**Conclusion:**

In this sample, regardless of the treatment previously received patients with or without diabetes and preserved ventricular function experienced similar outcomes. However, patients with ventricular dysfunction had a worse prognosis compared with those with normal ventricular function; patients with diabetes had greater mortality than patients without diabetes.

*Trial registration* http://www.controlled-trials.com. Registration Number: ISRCTN66068876

## Background

Large randomized trials have demonstrated consistently that patients with stable coronary artery disease (CAD) and preserved left ventricular function have a favorable clinical prognosis [1, 2]. Additionally, when different therapeutic strategies are compared, the prognosis continues to be quite good [3]. On the other hand, left ventricular dysfunction contributed to a poor prognosis during long-term follow-up [4]. Initial studies aimed at comparing drug therapy versus surgical revascularization in patients with left ventricular dysfunction in long-term follow-up revealed a worse prognosis for patients receiving drug therapy [5]. Later studies with the same objective failed to confirm the superiority of surgery over drug therapy [6]. Furthermore, in a partial analysis of the results of the BARI-Trial in diabetic patients with preserved ventricular function, surgical treatment was shown to have a superior impact on the prognosis of patients compared with percutaneous intervention [7]. However, in a subsequent study, BARI 2D, in exclusively diabetic patients, the same result could not be reproduced [8].

These studies investigated the efficacy of initial treatment with both percutaneous and surgical interventions in patients with or without ischemic cardiomyopathy. Therefore, they sought to gain knowledge about the role of interventional treatment in ventricular function. On the other hand, they were unable to evaluate the long-term prognosis of ventricular function after the surgical and percutaneous interventions.

Although the few comparative results confirm the burden of ventricular dysfunction in the long-term survival of coronary patients without diabetes, uncertainty remains about the prognosis of ventricular dysfunction in diabetic patients compared with nondiabetic patients. Additionally, there is a consensus that diabetes mellitus adds a higher degree of severity to this condition, and, when associated with chronic renal failure, the risk of premature death is definitively established. Trials aimed at studying each condition separately may find insurmountable methodological difficulties.

This study aimed to find results of long-term follow-up in outpatients included in a database of coronary disease patients who had previously received either medical treatment alone, or percutaneous coronary intervention, or coronary artery bypass surgery. In addition, the study aimed to compare whether preserved or compromised ventricular function had effects on diabetes mellitus.

## Methods

The database from the MASS Study group and REVISION-DM, at the Heart Institute of the University of São Paulo, includes CAD patients undergoing the 3 therapeutic options who received long-term follow-up. From this database, samples are available for randomized trials as well as “real-world” treatment assessments for follow-up. Thus, this database was important for supplying patients for the MASS-II Trial (MASS-II Registration Number SRCTN 660668876). For the current REVISION-DM study, patients with stable multivessel coronary disease with and without diabetes who had previously received one of the 3 therapeutic options were included sequentially, prospectively, and were followed quarterly until May 2015. Measurements of left ventricular ejection fraction were obtained for all patients during the inclusion process.

### Recruiting patients for registration

Study records were designed to include patients with stable CAD and documented myocardial ischemia for use in many trials. Thus, these patients formed a large database generating multiple analyzes. Patients with stable multiarterial coronary disease who had various therapeutic options available for CAD were considered for this study: medical, surgical, or percutaneous treatment. Myocardial ischemia was documented through a stress test or myocardial scintigraphy. Angina pectoris, when present, was graded by using Canadian Cardiovascular Society (CCS) classes II or III. For inclusion in this study, ventricular function was assessed by transthoracic echocardiography and LVEF using the Simpson method. LVEF was considered preserved when values were ≥ 55% and compromised when ≤ 35%. This analysis included patients with stable CAD, and optimized medical therapy alone, CABG, or PCI. Patients with limited life expectancy or incapacity for long-term outpatient follow-up were not included. In addition, we did not include patients with artificial cardiac devices or dialytic or cardiac transplant patients. Patients were considered to have diabetes if, at baseline, they were using insulin and/or oral hypoglycemic agents, of if they had the classical criteria for type 2 diabetes mellitus as stated by the American Diabetes Association [9] (2 fasting glucose measures ≥ 126 mg/dL, glycated hemoglobin [A1c] ≥ 6.5%, random glucose ≥ 200 mg/dL, or 2-h plasma glucose ≥ 200 mg/dL during an oral glucose tolerance test).

### Treatment protocol

The clinical treatment indicated for patients was medical therapy for the relief of angina symptoms and heart failure. For secondary prevention of cardiovascular events, therapeutic targets were used as recommended by the specific guidelines. The medications used included nitrates, acetylsalicylic acid, beta-blockers, calcium channel blockers, diuretics, spironolactone, angiotensin-converting enzyme inhibitors, statins, or a combination of these drugs. A diet low in saturated fats and carbohydrates was recommended. Insulin and oral hypoglycemic agents were prescribed for better control of hyperglycemia. For patients undergoing PCI, bare-metal or drug-eluting stents were used at the physician’s discretion. The interventional cardiologist was encouraged to perform complete revascularization. Angioplasty was performed according to the institutional protocols where acetylsalicylic acid and/or clopidogrel were prescribed before the procedure. Treatment with platelet antiaggregation after angioplasty followed the guidelines of national and international societies.

For the patients who underwent the surgical intervention, a complete and anatomic revascularization was planned. The use of the internal mammary artery, as a graft, was strongly recommended. The surgical procedure also complied with standardized techniques with the application of mild hypothermia and blood cardioplegia in patients operated on during extracorporeal circulation. Surgery without the extracorporeal circulation was performed according to medical criteria.

### Follow-up

The patients were followed regularly in periodic and semi-annual consultations for a rigorous clinical evaluation. Clinical events were recorded and dated from patient inclusion in the study. Laboratory tests to monitor therapeutic, lipid, and glycemic goals were requested semiannually. Echocardiography and subsidiary examinations to evaluate cardiac function were requested according to clinical indication.

### Outcomes

The events considered were overall mortality, nonfatal myocardial infarction, stroke, and additional interventions. The diagnosis of myocardial infarction was established when chest pain, new “Q” waves in 2 or more contiguous leads on the ECG, and elevated biomarkers of myocardial necrosis were present. Heart failure was diagnosed according to the presence of symptoms of dyspnea, pulmonary rales, tissue hypoperfusion, and peripheral edema. The American Heart Association guidelines were followed for the grading of heart failure [10].

### Statistical analysis

All data were analyzed immediately after patients were included in the study. Values are expressed as mean (± SD) or median (interquartile range 25–75%) as appropriate. Dichotomous data were compared using the χ^2^ statistic or Fisher’s exact test. The continuous variables that were not distributed normally were evaluated using the Kolmogorov–Smirnov test and compared with the Mann–Whitney test. Continuous variables with a normal distribution were compared using the Student *t* test. All reported probability values are 2-sided. Combined event-free survival was graphically compared and estimated by using the Kaplan–Meier method, and differences among groups were assessed using the log-rank test. Cox regression with model-robust standard errors (as implemented under the SAS PHREG routine) was used to compare survival time with combined primary end points and with each of the components of the primary end points among the different pairwise treatment groups. Multivariate analysis was also performed, adjusted for overall patients, age, sex, hypertension, past or present smoking status, triglycerides, total cholesterol level, and creatinine clearance (variables known to be related to poor outcomes). We performed subgroup analyses of assigned treatment with baseline characteristics using Cox regression. Tests were 2-tailed, and values of *P* < .05 were considered statistically significant, except for the treatment comparisons within identified subgroups, in which *P* < .01 was used to control for multiple comparisons. Statistical analyses were performed with SPSS software, version 24 (SPSS, IBM Corporation, Chicago, III).

## Results

### Characteristics of the patients and treatment assignments

**Fig. 1:**
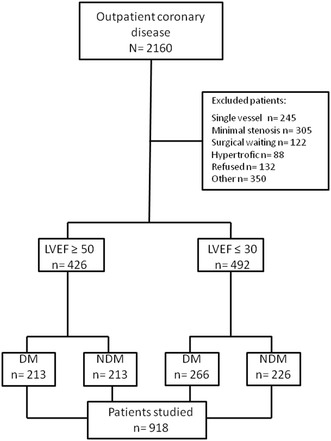
Number of patients assessed, enrolled, and included in the trial

Between January 2009 and May 2010, 2160 patients with CAD were selected. Of these, 918 patients were included in the study. They were included sequentially, prospectively, and followed quarterly until May 2015. The vital status of all included patients was ascertained in May 2015. For patients still alive, the minimum length of follow-up was 5 years, and the maximum was 6 years (average 5.3 years). According to ventricular function and the presence or absence of diabetes, 4 groups were selected (Fig. 1).

Of the 918 patients included in the study, 426 (46.4%) had preserved LVEF and 492 (53.6%) had ventricular dysfunction. Of those with preserved ventricular function, 50% had diabetes and formed Groups 2 and 3, and of those with ventricular dysfunction, 54% formed Groups 1 and 4.

The formation of the 4 groups created balanced treatment groups with respect to important prognostic characteristics for preserved ventricular function and ventricular dysfunction, as depicted in Table 1. That is, patients in all 4 groups were similar with respect to age, sex, employment status, past or present tobacco use, and hypertension. Severity of angina was similar in patients with preserved ventricular function, and heart failure was similar in patients with ventricular dysfunction. Patients assigned to the 4 groups were also similar in terms of proportional number of vessel disease and treatment previously received. All patients received optimal medical regimens per predefined guidelines.

**Table 1:** Demographic, clinical, laboratory, and angiographic characteristics

Patients	G1 LVEF ≤ 30% (n = 266)	G2 LVEF ≥ 55% (n = 213)	G3 LVEF ≥ 55% (n = 213)	G4 LVEF ≤ 30% (n = 226)	*χ* ^2^
Demographic profile %					
Age (years)	66	67	70	65	28.9*
Age ≥ 65 years	53	62	63	54	26.2*
Male	66	67	69	65	23.3*
Smokers or ex-smokers	57	58	60	55	10.4*
Medical history %					
Previous infarction	90	51	45	88	42.3^†^
Hypertension	67	62	65	68	96.8*
Diabetes mellitus	100	100	00	00	NA
CHF class I ou II	70	00	00	72	NA
Laboratory (mg/dL)					
Total cholesterol	226 ± 4	224 ± 6	216 ± 6	211 ± 3	48.6*
LDL cholesterol	138 ± 14	140 ± 12	136 ± 16	132 ± 11	62.2*
HDL cholesterol	39 ± 7	38 ± 8	36 ± 6	37 ± 8	44.5*
Triglycerides	170 ± 8	166 ± 10	172 ± 5	168 ± 7	68.3^‡^
Glycated hemoglobin (%)	6.8 ± 2	6.9 ± 3	5.8 ± 8	5.7 ± 9	NA
Creatinine clearance (mL/min)	34.5 ± 9	84.3 ± 11	78.4 ± 8	58.2 ± 9	59.6^‡^
Positive exercise test (%)	NA	68	65	NA	NA
Angiographic data (%)					
Two-vessel disease	29	32	46	31	35.7*
Three-vessel disease	71	68	64	69	48.2*
Ejection fraction (average)	30	58	60	29	NA

*G1* Group 1, *G2* Group 2, *G3* Group 3, *G4* Group 4, *LVEF* left ventricular ejection fraction, *CHF* congestive heart failure, *NA* not applicable

* *P* < .10, ^†^ *P* < .001, ^‡^ *P* < .05

### Preserved ventricular function

Groups 1 and 3 were composed of 426 patients with preserved ejection fraction, 213 of who had diabetes; 147 received medical treatment, 116 percutaneous intervention, and 163 surgical treatment. In the patients operated on, 3.1 ± 1.5 arterial or venous grafts per patient were performed, whereas in patients who underwent percutaneous interventions, 2.9 ± 1.5 obstructed stenoses per patient were treated.

### Ventricular dysfunction

Groups 1 and 4 comprised 492 patients with ventricular dysfunction, 266 of whom had diabetes; 134 received medical treatment, 145 percutaneous intervention, and 211 surgical treatment. In the patients operated on, 2.9 ± 1.4 arterial or venous grafts per patient were performed, whereas in patients who underwent percutaneous interventions, 2.7 ± 1.4 obstructed stenoses per patient were treated.

### Follow-up outcomes

The overall major adverse cardiac and cerebrovascular events at the 5-year follow-up per ventricular function are shown in Table 2. No patients from any of the 4 study groups were lost to follow-up.

**Table 2:** Major adverse cardiac events at 5-year follow-up

Patients	Group 1 (LVEF ≤ 30)	Group2 (LVEF ≥ 55)	Group3 (LVEF ≥ 55)	Group4 (LVEF ≤ 30)	χ^2^
Nonfatal AMI	12	1	15	6	14.11*
Overall mortality	49	13	9	24	31.48*
Stroke	1	1	2	3	1.81
Intervention	10	25	22	6	22.19*

*LVEF* left ventricular ejection fraction, *AMI* acute myocardial infarction, *Intervention* surgical or percutaneous

* *P* < .001

### Event-free survival

**Fig. 2:**
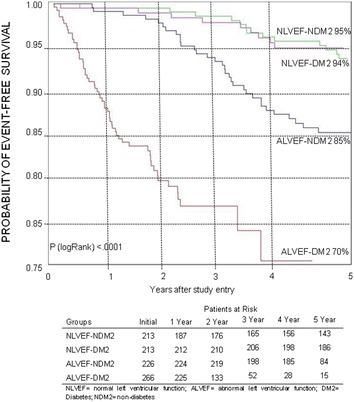
Probability of survival free of overall mortality, unstable angina requiring revascularization, and myocardial infarction and stroke among patients in the groups

The rates of event-free survival, namely the combined incidence of overall mortality, nonfatal MI, stroke, or refractory angina that required revascularization, were significantly different among patients in the 4 groups studied in the 5-year follow-up (*P* < .01) (Fig. 2).

Pairwise treatment comparisons of the major adverse cardiac events at 5-year follow-up showed a significant difference between patients with preserved ventricular function and those with ventricular dysfunction (hazard ratio [HR] 5.26, 95% CI 2.03–13.6). In addition, when we compared diabetic and nondiabetic patients with preserved ventricular function, we did not observe differences (HR 1.19, 95% CI .69–2.05). On the other hand, when comparing diabetic and nondiabetic patients with ventricular dysfunction, we observed statistically significant differences (HR .38, 95% CI .24–.59).

**Fig. 3:**
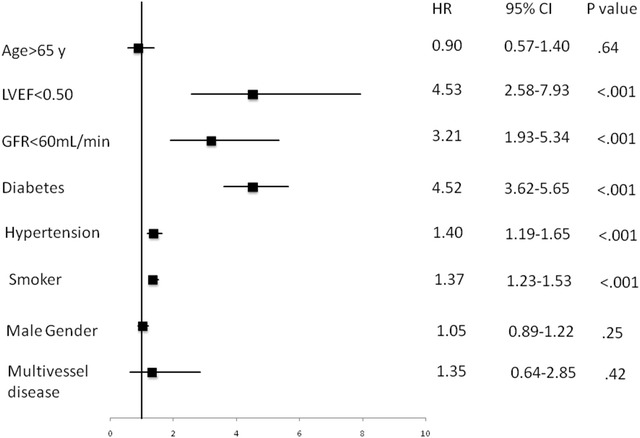
Cox-proportional hazards regression model for ventricular dysfunction versus preserved ventricular function

After using the Cox proportional hazard model at 5-year follow-up, we observed a strong interference in the prognosis of LVEF (HR .01, 95% CI .05–.02, *P* < .001), creatinine clearance (HR .99, 95% CI .91–1.07, *P* < .001), diabetes mellitus (HR 4.25, 95% CI 3.62–5.65, *P* < .001), hypertension (HR 1.40, 95% CI 1.19–1.65, *P* < .001), and tobacco use (HR 1.37, 95% CI 1.23–1.53, *P* < .001). We did not find differences between age, sex, and number of diseased vessels (Fig. 3).

**Fig. 4:**
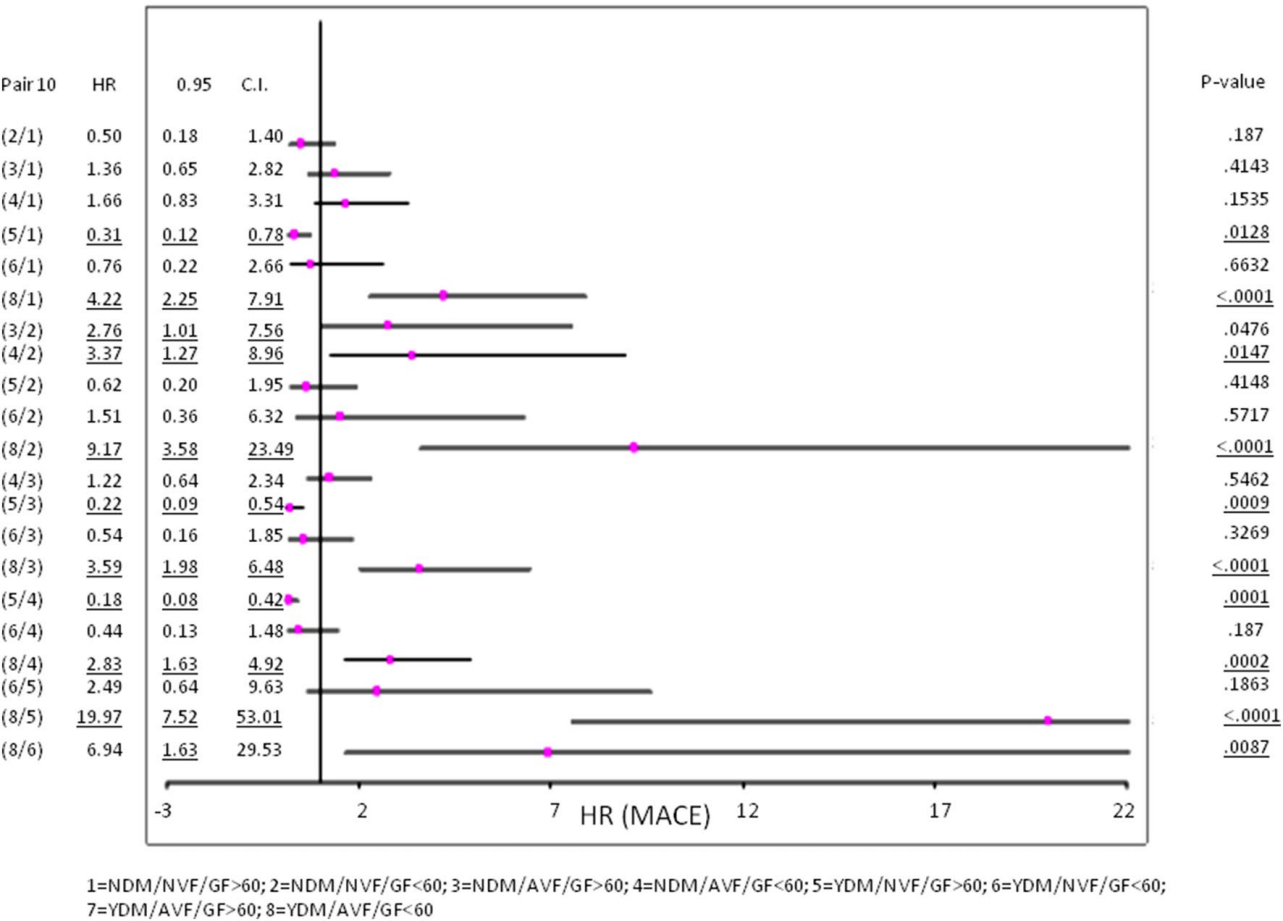
Relationship between diabetes ventricular function and glomerular filtration (dichotomized 60 mL/min)

Furthermore, when performing the logistic regression analysis, we found that ventricular dysfunction, diabetes, and glomerular filtration made the prognosis worse in this study sample (Fig. 4).

### Overall mortality

**Fig. 5:**
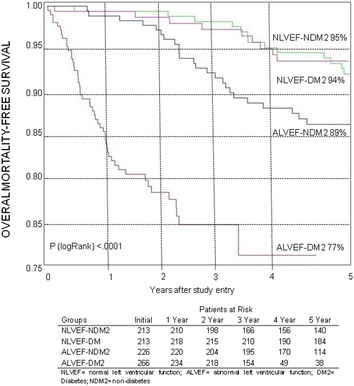
Probability of survival free of overall mortality among patients

No significant statistical differences existed among the cumulative overall mortality curves associated with groups with preserved ventricular function with or without diabetes. However, in patients with ventricular dysfunction, we observed significant statistical differences compared with patients with preserved ventricular function (*P* < .001). Additionally, in those with ventricular dysfunction, diabetic patients had significantly increased mortality (*P* < .001). Furthermore, comparing patients, both with ventricular dysfunction with and without diabetes, a higher mortality was found in those with diabetes (*P* < .001). The cumulative survival rates at 5-year follow-up for patients assigned to each group were 94% for Group 2, 95% for Group 3, 89% for Group 4, and 77% for Group 1 (Fig. 5).

### Analysis of groups

The presence of diabetes at 5-year event-free survival was not changed by preserved ventricular function. However, the occurrence of events was strongly apparent in patients with diabetes and ventricular dysfunction (Fig. 2).

We were not able to detect in diabetic patients any additional risk factors except ventricular dysfunction. However, in those with dysfunction, the diabetes patients had a worse outcome. In addition, significantly lower creatinine clearance appears to have contributed to the poorer prognosis in the patients with diabetes. It remains to be seen whether the worse glomerular filtration was the cause or effect of the worse prognosis (Fig. 3).

## Discussion

The results of the present study revealed that patients with preserved ventricular function have a similar occurrence of cardiovascular events, independently of the presence of diabetes.

On the other hand, compared with patients with impaired left ventricular function, there was a significant increase in events and general mortality. Moreover, the presence of diabetes mellitus in patients caused increased events and death compared to patients with dysfunction and without diabetes.

In fact, the occurrence of combined events between patients with and without diabetes and preserved ventricular function was not significantly statistically different (*P* < .888), whereas a worse outcome was observed in the presence of ventricular dysfunction (*P* < .001). Furthermore, a worse result was observed in diabetic patients compared with nondiabetic patients, both with ventricular dysfunction (*P* < .001). Results of the STICH trial [6] regarding ventricular dysfunction revealed an annual mortality rate of 8%, whereas in the clinical arm of the CASS Trial [11] mortality was 11%. Annual mortality in our study reached 10.5% in nondiabetic patients and 18.4% in diabetic patients with ventricular dysfunction. This result reveals the importance of diabetes in the mortality of patients with ventricular dysfunction. The concept of diabetic cardiomyopathy, based on postmortem findings, [12] allowed advances in the knowledge of left ventricular systolic and diastolic damage. Subsequent studies have indicated that left ventricular diastolic dysfunction represents the earliest preclinical manifestation of diabetic cardiomyopathy, preceding systolic dysfunction, and that it progresses to symptomatic heart failure [13, 14]. These studies allowed us to infer that important mechanisms of diabetic cardiomyopathy are attributed to metabolic disturbances; myocardial fibrosis, such as increases in angiotensin II, IGF-inflammatory cytokines; and small vessel disease, for example microangiopathy, and endothelial dysfunction. Additionally, such mechanisms may play an important role in cardiac autonomic neuropathy and insulin resistance. These mechanisms can operate in association or in isolation to a greater or lesser degree, or even not interfere in the evolution of the disease [15].

In this context, the adverse effects of diabetes mellitus in our study were perceptible in patients with ventricular dysfunction. In those with preserved function, these changes remained “unperceived.”

Another factor that interfered with the worsening of mortality in our study was renal function. Associated with diabetes, renal failure was a determining factor of worse prognosis in patients with ventricular dysfunction. In fact, the SOLVD-study, which included only patients with ventricular dysfunction but without identifying diabetic patients, reported that there was a statistically significant interaction (*P* = .022) between predicted glomerular filtration rate and all-cause mortality. Thus, the lower level of the glomerular filtration rate was associated with higher all-cause mortality than expected from the sum of the individual effects [16, 17] Our study confirms the influence of renal function on the worse prognosis of patients with ventricular dysfunction. In addition, study results suggest that the association of diabetes with lower glomerular filtration rate strongly interferes with the mortality of these patients.

In this backdrop, the noninterference of diabetes in patients with normal ventricular function clearly contrasted with the worse prognosis in those with ventricular dysfunction. Another complicating factor, chronic kidney disease, contributed to a worse prognosis in this group. In this scenario, it remains to be seen whether chronic kidney disease was the cause or effect of this worse prognosis. Additionally due to the retrospective nature of the study, fine controls of Hb A1c sequences were not made. Large-scale studies aimed at answering this question are needed.

## Conclusion

In this sample, regardless of the treatment previously received patients with or without diabetes and preserved ventricular function experienced similar outcomes. However, patients with ventricular dysfunction had a worse prognosis compared with those with normal ventricular function; patients with diabetes had greater mortality than patients without diabetes.

## Abbreviations

CABG: coronary artery bypass graft
CAD: coronary artery disease
LVEF: left ventricular ejection fraction
MT: medical therapy
PCI: percutaneous coronary intervention

## References

[1] Boden WE, O’Rourke RA, Teo KK, Hartigan PM, Maron DJ, Kostuk WJ (2007). Optimal medical therapy with or without PCI for stable coronary disease. N Engl J Med.

[2] Henderson RA, Pocock SJ, Clayton TC, Knight R, Fox KA, Julian DG (2003). Seven-year outcome in the RITA-2 trial: coronary angioplasty versus medical therapy. Second randomized intervention treatment of Angina (RITA-2) trial participants. J Am Coll Cardiol.

[3] Iqbal J, Zhang YJ, Holmes DR, Morice MC, Mack MJ, Kappetein AP (2015). Optimal medical therapy improves clinical outcomes in patients undergoing revascularization with percutaneous coronary intervention or coronary artery bypass grafting: insights from the Synergy Between Percutaneous Coronary Intervention with TAXUS and Cardiac Surgery (SYNTAX) trial at the 5-year follow-up. Circulation.

[4] Zannad F, Garcia AA, Anker SD, Armstrong PW, Calvo G, Cleland JG (2013). Clinical outcome endpoints in heart failure trials: a European Society of Cardiology Heart Failure Association consensus document. Eur J Heart Fail.

[5] Passamani E, Davis KB, Gillespie MJ, Killip T, The CASS Principal Investigators and Their Associates (1985). A randomized trial of coronary artery bypass surgery: survival of patients with a low ejection fraction. N Engl J Med.

[6] Velazquez EJ, Lee KL, Deja MA, Jain A, Sopko G, Marchenko A (2011). Coronary-artery bypass surgery in patients with left ventricular dysfunction. N Engl J Med.

[7] The Bypass Angioplasty Revascularization Investigation (BARI) Investigators (1996). Comparison of coronary bypass surgery with angioplasty in patients with multivessel disease. N Engl J Med.

[8] Frye RL, August P, Brooks MM, Hardison RM, Kelsey SF, MacGregor JM (2009). A randomized trial of therapies for type 2 diabetes and coronary artery disease. BARI 2D study group. N Engl J Med.

[9] American Diabetes Association (2011). Diagnosis and classification of diabetes mellitus. Diabetes Care.

[10] Hunt SA (2005). American College of Cardiology; American Heart Association task force on practice guidelines (Writing Committee to Update the 2001 guidelines for the evaluation and management of heart failure). ACC/AHA 2005 guideline update for the diagnosis and management of chronic heart failure in the adult: a report of the American College of Cardiology/American Heart Association task force on practice guidelines (Writing Committee to Update the 2001 guidelines for the evaluation and management of heart failure). J Am Coll Cardiol.

[11] Passamani E, Davis KB, Gillespie MJ, Killip T (1985). A randomized trial of coronary artery bypass surgery. Survival of patients with a low ejection fraction. N Engl J Med.

[12] Rubler S, Dlugash J, Yuceoglu YZ, Kumral T, Branwood AW, Grishman A (1972). New type of cardiomyopathy associated with diabetic glomerulosclerosis. Am J Cardiol.

[13] Raev DC (1994). Which left ventricular function is impaired earlier in the evolution of diabetic cardiomyopathy? An echocardiographic study of young type I diabetic patients. Diabetes Care.

[14] Bell DS (1995). Diabetic cardiomyopathy. A unique entity or a complication of coronary artery disease?. Diabetes Care.

[15] Fang ZY, Prins JB, Marwick TH (2004). Diabetic cardiomyopathy: evidence, mechanisms, and therapeutic implications. Endocr Rev.

[16] Al-Ahmad A, Rand WM, Manjunath G, Konstam MA, Salem DN, Levey AS (2001). Reduced kidney function and anemia as risk factors for mortality in patients with left ventricular dysfunction. J Am Coll Cardiol.

[17] Weiner DE, Tighiouart H, Amin MG, Stark PC, MacLeod B, Griffith JL (2004). Chronic kidney disease as a risk factor for cardiovascular disease and all-cause mortality: a pooled analysis of community-based studies. J Am SocNephrol..

